# Local Oxygen-Based Therapy (blue^®^m) for Treatment of Peri-Implant Disease: Clinical Case Presentation and Review of Literature about Conventional Local Adjunct Therapies

**DOI:** 10.3390/medicina60030447

**Published:** 2024-03-08

**Authors:** Marwa Y. Shaheen, Irfan Abas, Amani M. Basudan, Hamdan S. Alghamdi

**Affiliations:** 1 Department of Periodontics and Community Dentistry, College of Dentistry, King Saud University, P.O. Box 2455, Riyadh 11451, Saudi Arabia; abasudan@ksu.edu.sa (A.M.B.); dalghamdi@ksu.edu.sa (H.S.A.); 2 Department of Oral Implantology and Restorative Dentistry, Academy and Private Practice, Herenstraat 37, 1404 HC Bussum, The Netherlands; irfan.abas@gmail.com

**Keywords:** dental implant, topical administration, oxygen therapy, peri-implant mucositis, peri-implantitis

## Abstract

Peri-implant diseases including peri-implant mucositis and peri-implantitis are among the major causes of failure of implant-supported dental restorations. They are characterized by progressive inflammation of the peri-implant mucosa, extending to the surrounding connective tissues and leading to bone loss and implant failure. Although strict oral hygiene practices help in preventing peri-implant diseases, plaque buildup around the implant restoration leads to chronic inflammation, due to the adherent bacterial biofilm. While mechanical debridement and non-surgical therapy to remove inflamed connective tissue (ICT) form the mainstay of treatment, additional local adjunctive therapies enhance clinical outcomes. Topical oxygen therapy is known to reduce inflammation, increase vascularity, and act as a bacteriostatic measure. The use of oxygen-based therapy (blue^®^m) products as a local adjunctive therapy for peri-implant mucositis and peri-implantitis can result in clinical outcomes similar to that of conventional local adjuncts such as chlorhexidine, antibiotics, and antibacterial agents. This report aims to present the clinical findings of patients with peri-implant mucositis and peri-implantitis, who were managed using local oxygen-based therapy as an adjunct to non-surgical therapy. In addition, a review of the literature about commonly used local adjuncts for peri-implant diseases has been included in the report to provide a means of comparison between conventional local adjunct therapy and topical oxygen-based therapy. Based on the reported findings and reviewed literature, local oxygen-based adjunct therapy was equally effective as conventionally used local adjuncts such as antibiotics, antibacterials, and probiotics, in treating patients with peri-implant diseases.

## 1. Introduction

Peri-implant diseases including peri-implant mucositis (PIM) and peri-implantitis (PI) are among the major causes of failure of implant-supported dental restorations [1]. According to the “Consensus report of workgroup 4 of the 2017 World Workshop on the Classification of Periodontal and Peri-Implant Diseases and Conditions”, and as per “EFP S3 level clinical practice guideline”, peri-implant diseases are defined as, “inflammatory conditions that affect the peri-implant tissues and are induced by peri-implant biofilms” [2,3]. While PIM is similar to gingivitis and is characterized by inflammation of the mucosa surrounding the implant, PI is a progressive inflammatory condition like periodontitis and involves the surrounding connective tissue and supporting bone [1]. In most clinical scenarios, PIM without proper intervention progresses to PI, leading to inflammatory connective tissue (ICT) and bone loss, and ultimately failure of the implant [4]. Peri-implant disease can be defined as a pathological entity associated with the buildup of plaque biofilm around the implant and restoration, and the importance of meticulous oral hygiene in disease prevention is reinforced by the fact that their discontinuation for even a few days to weeks could lead to PIM [5]. It has been shown that, similar to gingivitis and periodontitis, plaque biofilm surrounding the implant is capable of inducing clinical, histological, and immunological changes in the peri-implant tissues [5]. While clinical changes include inflammatory signs such as redness, swelling, bleeding on probing (BOP), and suppuration, inflammatory infiltration with pro-inflammatory cytokines and pocket formation characterize the histological and immunological spectrums of the disease [4,5].

Based on reported case definitions and diagnostic criteria, PIM can be clinically confirmed by the presence of BOP, signs of inflammation including redness and swelling, and probing pocket depth (PPD) less than 3–5 mm [4]. Similarly, PI is diagnosed when there is PPD greater than/equal to 5 mm in association with radiographic evidence of bone loss either greater than 2 mm or more than two implant threads. Additionally, the peri-implant soft tissues may present with BOP and/or suppuration at more than one site [4]. In general, PIM is reversible upon removal of the bacterial plaque biofilm through non-surgical mechanical debridement and home-based oral hygiene practices [4]. The commonly advocated non-surgical therapeutic means for PIM include professionally administered ultrasonic scaling with specialized piezo-ceramic tips [6], and sub-mucosal curettage with Teflon-, carbon-fiber-, or plastic-tipped hand instruments [7,8]. While the above non-surgical debridement procedures may also be advocated in cases of PI, additional sub-gingival debridement with specialized tips is performed on the implant surface [4]. This could further be accentuated by using site-specific air abrasion and polishing, using either amino acid glycine or erythritol powders [9,10,11,12]. Nevertheless, surgical approaches such as open flap debridement, apically repositioned flaps with implantoplasty, and regenerative procedures for soft tissue and bone have been clinically advocated in the management of PI [4]. Irrespective of the method of mechanical debridement for removal of plaque, calculus, and ICT, personal oral hygiene practices including brushing, mouth rinsing, and interdental plaque control are mandatory for peri-implant disease management [5].

The use of local adjunctive therapies has helped significantly alter the outcomes of non-surgical and surgical therapies for peri-implant disease [1,4,13,14]. It is especially alluring to hypothesize that the use of local adjuncts along with non-surgical therapy for PIM and PI would improve patient compliance and adherence to treatment protocols. Based on a systematic review, Ramanauskaite et al. [1] reported a reduction in BOP and PPD, enhanced peri-implant bone gain, and reduced gingival recession when local adjunctive measures were administered along with non-surgical therapy for peri-implant disease [1]. Clinically, several local and topical adjuncts have been reported for the treatment of both PIM and PI. Although these include several antibiotics, antibacterials, and probiotics [8,11,15,16,17], the most commonly used agent is chlorhexidine either as a mouthwash or as a gel [18,19,20]. The mechanism of action of chlorhexidine when used as a topical adjunct against plaque biofilm is through bacterial lysis after ionic attachment to its cell surface [21]. While this may contribute to its antibacterial efficacy, the chlorhexidine molecule due to its large size lacks the ability to penetrate and modulate the biofilm, for even better action [21,22]. The same scenario applies to other antibiotics and antibacterial agents reportedly used as topical adjuncts [13,14]. Therefore, these local adjuncts do not contribute to the complete clinical resolution of peri-implant diseases [1,5]. Moreover, these chemical agents are liable to induce hypersensitivity and adverse effects when used in sensitive individuals and for the long term, respectively [22].

In the last few years, an oxygen-based local adjunct formulation (blue^®^m) was developed by Peter Blijdorp and colleagues [23], for use in the treatment of periodontal and peri-implant diseases. Accordingly, these oxygen-based therapy products have been clinically used as an oral gel, mouthwash, and toothpaste with considerably better treatment outcomes [23,24,25]. Composed primarily of sodium peroxoborate, glycerol, lactoferrin, and cellulose, the blue^®^m oxygen-based formulations are capable of slow and sustained oxygen release, when applied topically. This helps in wound bio-modulation, reduced inflammation, enhanced healing, and neovascularization [23]. In addition, they also release hydrogen peroxide with lactoferrin, which bestows bactericidal benefits [25]. Based on the recommendations of Dr. Peter Blijdorp and colleagues, local oxygen-based therapy may be administered using the TOOTh (Topical oral oxygen therapy) protocol and blue^®^m formulations (Bluem Europe Inc., Enkweg, Wijhe, The Netherlands; oral gel, toothpaste, and mouthwash) [25]. Accordingly, the protocol includes initial clinical and radiographic assessment followed by professionally administered scaling and non-surgical peri-implant cleaning and debridement [26]. At the same time of the procedure, blue^®^m oral gel would be administered sub-gingivally and the patient discharged home with instructions to brush twice daily and rinse with mouthwash for one minute [24]. Thereafter, the patient would perform self-administration of oral gel at the peri-implant site using an interdental brush. This process would be repeated three times at intervals of two weeks, until the patient comes for recall [24,25].

The present study aims to report a set of clinical cases with peri-implant diseases (PIM and PI), which were effectively managed using non-surgical therapy along with application of local oxygen-based therapy using the TOOTh protocol and blue^®^m formulations, and attained clinical disease resolution. In addition, this report reviews the pertinent literature about local adjunct therapies, in an effort to propose a protocol for the use of local oxygen-based formulations and non-surgical therapy as the preferred treatment modality for peri-implant diseases.

## 2. Clinical Case Presentation of Peri-Implant Mucositis

**Figure 1:**
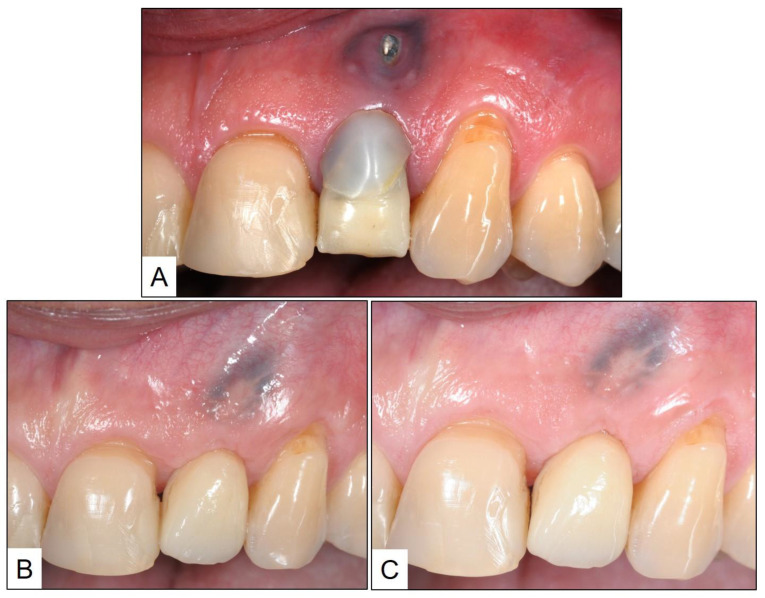
Pre- and post-treatment clinical presentation: (**A**) Initial examination, showing failed restoration in #22, with fractured crown and metallic post perforating the buccal gingiva, apical to gingival margin; (**B**) Immediate post-treatment image showing implant-supported crown, along with guided bone regeneration and soft-tissue augmentation; and (**C**) One-year post-treatment follow-up image showing healthy peri-implant mucosa, with no clinical signs of inflammation.

A healthy 33-year-old male patient reported to the clinic with failed restorative treatment in tooth #22 (maxillary left lateral incisor). The patient reported no remarkable medical, family, or social history and was a non-smoker. Apparently, the tooth was restored with a crown supported by a metallic post, placed after endodontic treatment several years ago. At the time of clinical presentation, the patient had a fractured crown with buccal perforation apical to the gingival margin, due to the failed post. The tooth was considered hopeless, and the patient was advised to have an extraction of tooth #22, followed by a dental implant along with guided bone regeneration (GBR) of peri-implant bone and sub-epithelial connective tissue graft (SCTG) for soft tissue augmentation. The second stage implant surgery was planned three months after implant placement, and four weeks after that, a screw-retained crown (lithium-di-silicate crown with titanium abutment) was delivered. The patient was followed up after one year, during which time, the peri-implant mucosa was healthy with no clinical signs of inflammation (PPD ≥ 3 mm, BOP) (Figure 1).

**Figure 2:**
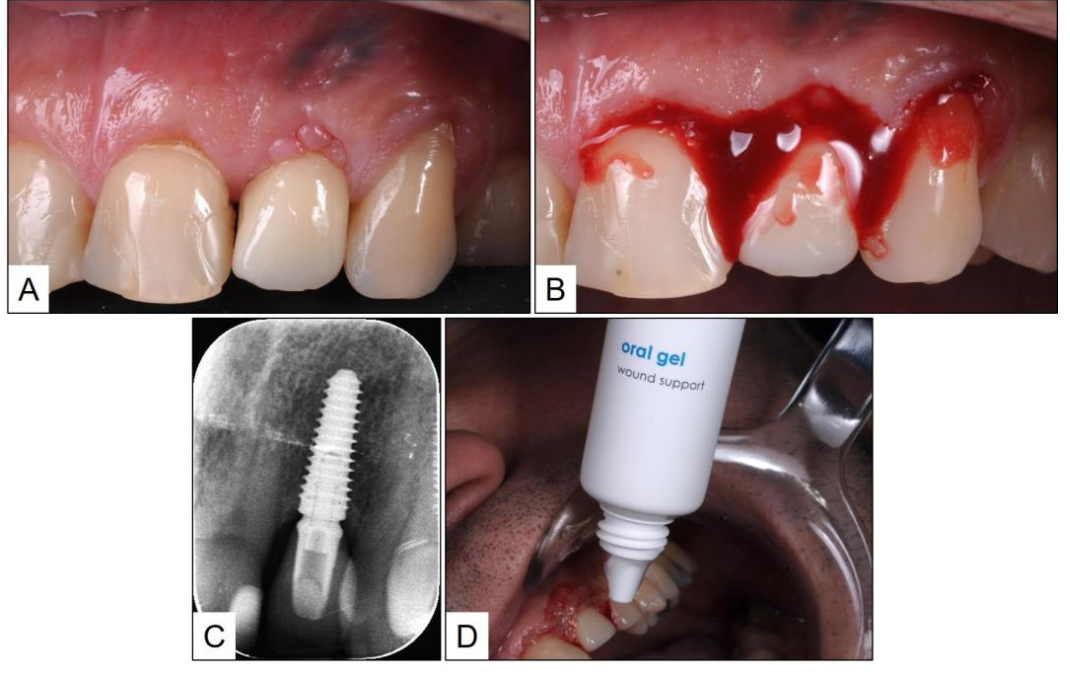
After two years of crown delivery: (**A**) Patient presented with marginal redness of the peri-implant mucosa in #22 area; (**B**) Clinically there was severe bleeding on probing; (**C**) Periapical radiographs revealed marginal peri-implant bone level at the same height as implant shoulder; and (**D**) Topical application of blue^®^m oral as part of treatment using TOOTh protocol.

One year after the last follow-up, which was chronologically two years after crown delivery, the patient presented to the clinic with marginal redness of the peri-implant mucosa. Upon examination, there were positive clinical signs of inflammation including a PPD of 5 mm and severe BOP. A periapical radiograph of the implant in #22 revealed a marginal peri-implant bone level at the same height as the implant shoulder. Since there was no radiographic evidence of bone loss, a clinical diagnosis of peri-implant mucositis was arrived at, and treatment was initiated as per the TOOTh protocol. Accordingly, non-surgical debridement of the peri-implant mucosa was performed under local anesthesia (LA) with an ultrasonic scaler and plastic curettes. This was followed by the reinforcement of meticulous oral hygiene using local oxygen therapy (blue^®^m) toothpaste and mouthwash, and topical application of blue^®^m oral gel, twice a day after cleaning. The patient was instructed not to spit or rinse for one hour after gel application (Figure 2).

**Figure 3:**
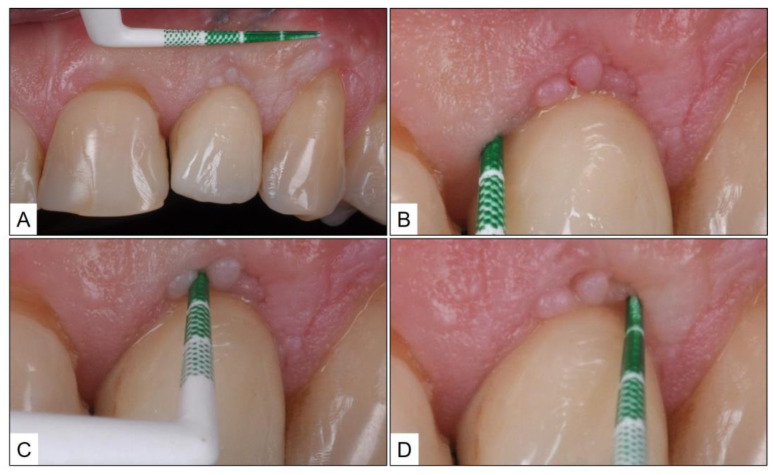
Eight weeks after initiation of TOOTh protocol: (**A**) Healthy peri-implant mucosa with no marginal redness or swelling; (**B**–**D**) No bleeding on probing and probing pocket depth ≤ 3 mm.

Eight weeks after treatment, the patient was followed up in the clinic. Upon examination, the peri-implant mucosa appeared healthy with no marginal redness, swelling, or suppuration. In addition, there was no BOP, and PPD was not more than 3 mm (Figure 3). The patient was advised to continue with the oral hygiene instructions and topical therapy with blue^®^m oral gel, on a daily basis.

## 3. Clinical Cases Presentation of Peri-Implantitis

### 3.1. Case Report 1

**Figure 4:**
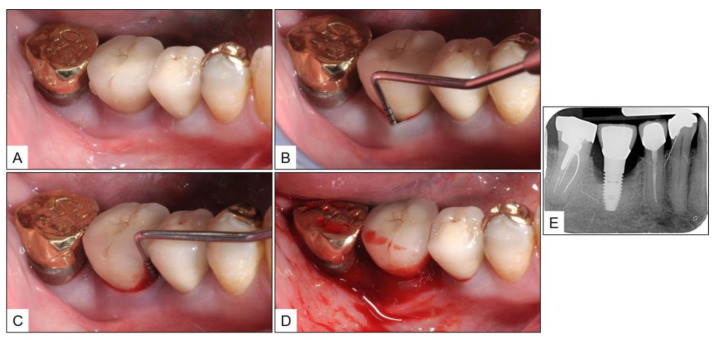
Patient referred with peri-implant disease in the #46 area: (**A**–**D**) Inflamed peri-implant mucosa with probing pocket depth up to 7 mm and severe bleeding on probing; and (**E**) Periapical radiograph showing bone loss around the implant; with bone margin level at the middle third of the implant.

A 67-year-old female patient with no remarkable medical, family, or social history was referred to the periodontal surgery clinic by a general dentist, for peri-implant disease in tooth #46 (mandibular right first molar) area. The general dentist had noticed bone loss around the implant with suppuration and, hence, the referral. Upon initial examination, there was severe BOP with pus discharge and PPD up to 7 mm. Periapical radiograph revealed bone loss around the implant, with the peri-implant bone margin almost at the level of the middle third of the implant (Figure 4).

Based on the clinical and radiographic findings, a diagnosis of peri-implantitis was arrived at. After consultation with the patient, it was decided to follow treatment as per the TOOTh protocol. Accordingly, non-surgical cleaning and mechanical debridement of the peri-implant soft tissue and implant surface was performed using an ultrasonic scaler with plastic tips and specialized Teflon-coated hand instruments. The non-surgical therapy was performed under LA and focused on the removal of plaque, calculus, and inflamed connective tissue (ICT). In addition, local oxygen-based therapy (blue^®^m) oral gel was injected at the peri-implant sub-mucosal area at the same time. The above procedure was repeated once every two weeks, and during the intervening post-operative periods, the patient was advised to practice oral hygiene twice a day using blue^®^m toothpaste and mouthwash, and apply blue^®^m oral gel around the peri-implant mucosa using an interdental brush, three times a day. The patient was instructed not to rinse or spit for one hour after application of oral gel, and was given follow-up appointments on second, fourth, and sixth weeks.

**Figure 5:**
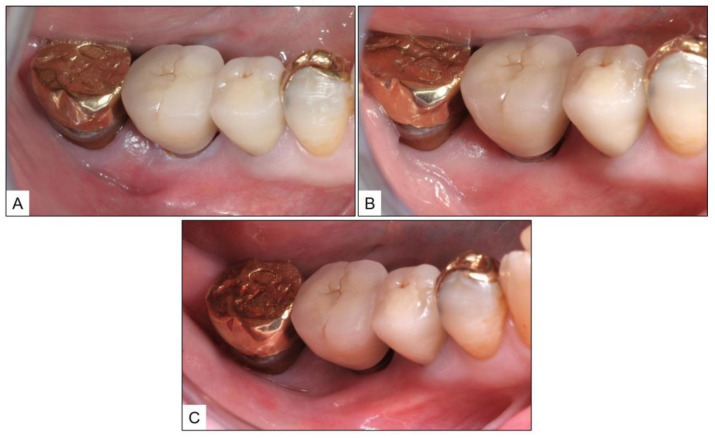
Clinical presentation of the peri-implant site during follow-up visits; showing decrease in the signs of soft-tissue inflammation: (**A**) Second week; (**B**) Fourth week; and (**C**) Sixth week.

**Figure 6:**
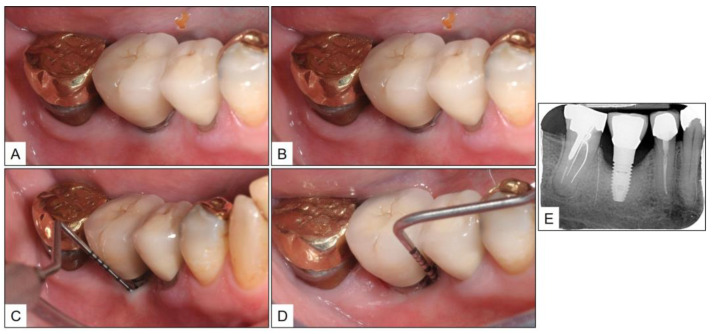
Clinical presentation of the peri-implant site three months after the final session of non-surgical therapy; showing resolution of inflammation: (**A**,**B**) No marginal swelling in spite of up to 2 mm gingival recession; (**C**) No bleeding on probing; (**D**) Reduction in probing pocket depth up to 3 mm; and (**E**) Periapical radiograph showing bone level gain by approximately two implant threads.

There was a progressive decrease in the peri-implant marginal swelling and BOP during the biweekly follow-up visits (Figure 5). While the patient complained about BOP at the time of brushing, during the second-week follow-up, the same was resolved by the time the patient reported for subsequent visits. Three months after the final session of non-surgical therapy (fourth week after the start of treatment), the patient was re-evaluated for PPD, BOP, and presence of suppuration, along with a new periapical radiograph. Although there was clinical evidence of gingival recession up to 2 mm, there was no BOP, and PPD was reduced by 3 mm. Similarly, there was a gain in radiographic bone level (RBL) up to approximately two implant threads (Figure 6).

### 3.2. Case Report 2

**Figure 7:**
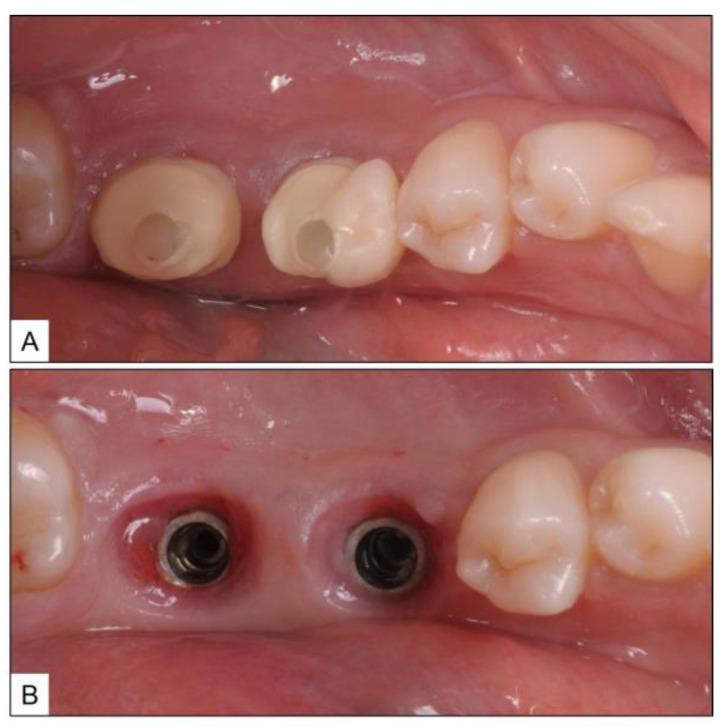
Pre-treatment clinical photographs showing (**A**) Fractured ceramic crowns supported by implants placed in the #36 and #37 areas; (**B**) Inflamed connective tissue is seen around the peri-implant areas of #36 and #37.

**Figure 8:**
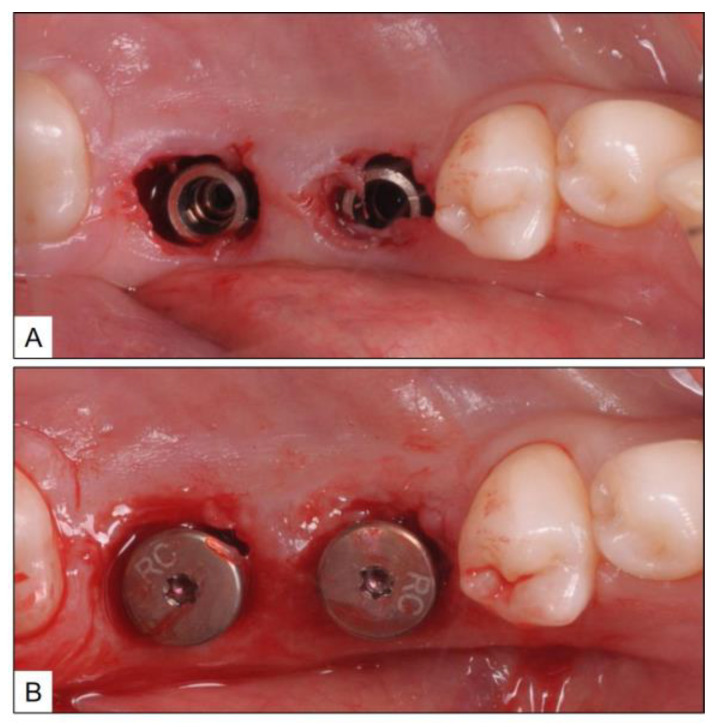
Immediate post-treatment clinical photographs showing (**A**) Debridement of inflamed connective tissue around the implants; (**B**) Placement of healing abutments after sub-gingival administration of oxygen-based therapy blue^®^m oral gel.

A healthy 44-year-old female patient was referred to the clinic for the replacement of fractured, implant-supported crowns in teeth #36 and #37 (mandibular left first and second molars) areas. Upon clinical examination, in addition to fractured ceramic crowns, there was also BOP with suppuration and PPD up to 7 mm, in both the implants (Figure 7). After consultation with the patient, the fractured crowns were removed and peri-implant disease treatment was initiated using local oxygen-based therapy as per the TOOTh protocol (blue^®^m). During the treatment phase, it was agreed not to restore the implants temporarily and to use healing abutments instead. Accordingly, the patient underwent full-mouth ultrasonic scaling and non-surgical mechanical debridement, using specialized tips, around the implants for removal of plaque, calculus, and ICT. This was followed by the sub-gingival application of blue^®^m oral gel, placement of healing abutment, and discharging the patient with advice to use blue^®^m toothpaste and mouthwash, twice a day, and subsequently by oral gel application using an interdental brush (Figure 8).

**Figure 9:**
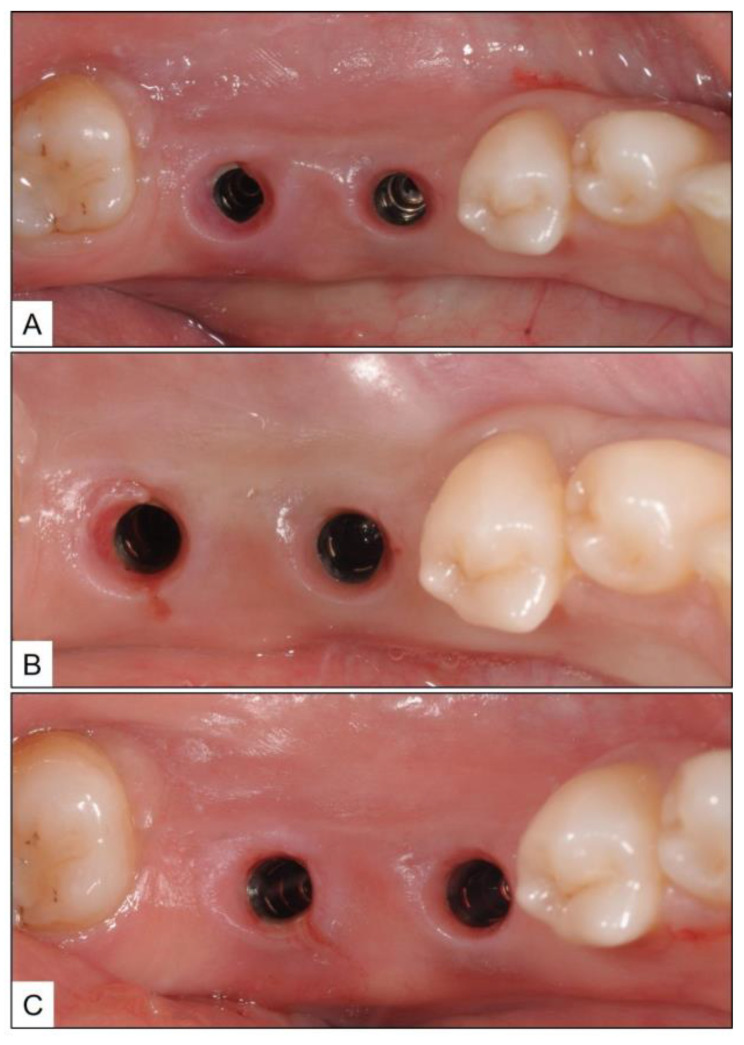
Follow-up clinical photographs showing reduction in peri-implant mucosal inflammation during (**A**) Second week; (**B**) Fourth week; and (**C**) Sixth week.

**Figure 10:**
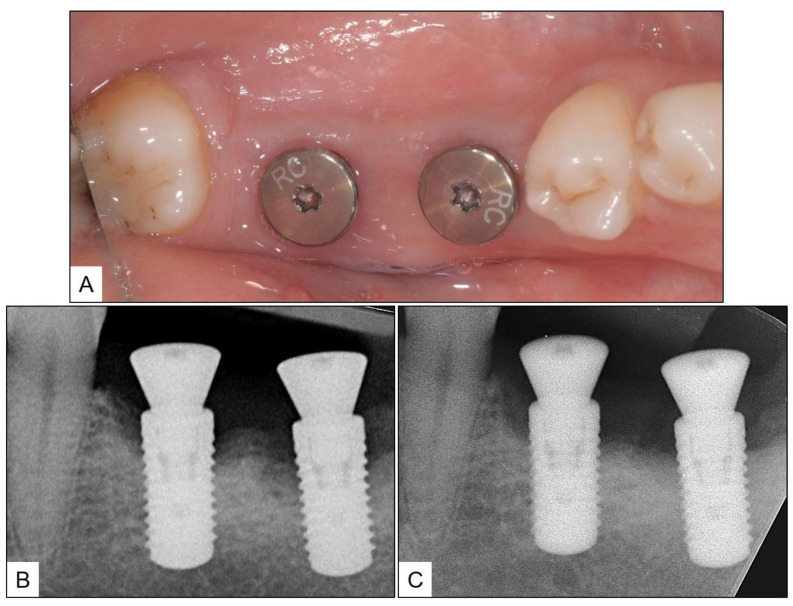
Follow-up clinical photograph after 3 months and comparison of radiographic bone height showing (**A**) Complete clinical resolution of peri-implantitis; 3 months after the last session of non-surgical therapy and local oxygen-based therapy with blue^®^m products; (**B**) Pre-treatment periapical radiograph showing peri-implant bone loss; and (**C**) Post-treatment periapical radiograph showing significant improvement in peri-implant bone height.

In all instances, the patients were further advised not to rinse or spit the mouth for an hour after oral gel application, and the aforementioned non-surgical therapy procedures were repeated on the second and fourth week. During the recall visits at two, four, and six weeks, the peri-implant mucosa showed a significant reduction in the clinical signs of inflammation (swelling and redness) (Figure 9). A further follow-up evaluation after three months from the last session of non-surgical therapy showed a complete clinical resolution of peri-implantitis, with no BOP or suppuration, and a reduction in PPD up to 3 mm. A periapical radiograph taken at the time showed a significant improvement in RBL when compared to the pre-treatment radiographic record (Figure 10).

## 4. Systematic Review of Literature—Methodology and Results

**Figure 11:**
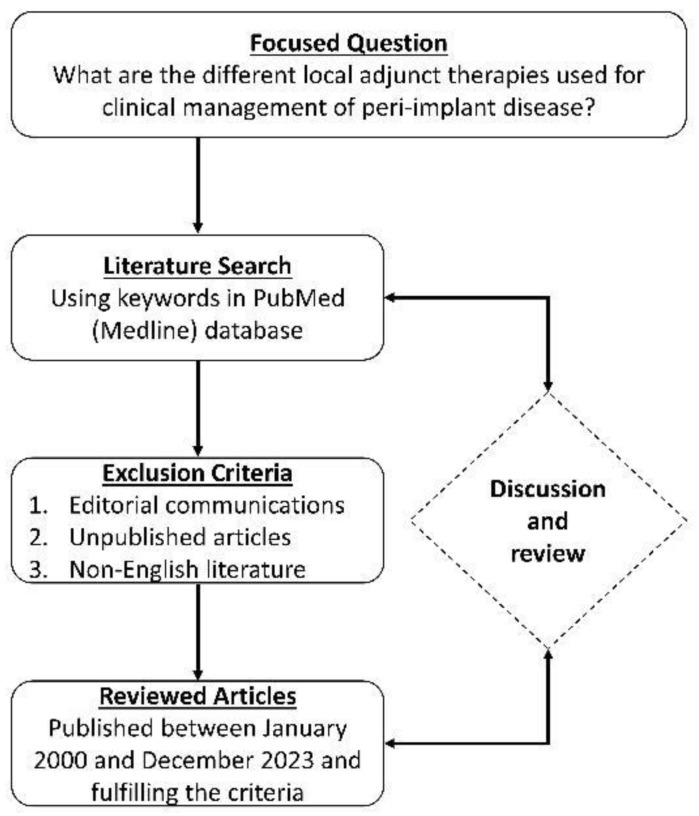
Flowchart for literature search and review.

A database search of scientific literature published in English was conducted by searching PubMed (Medline), with the focused question, “What are the different local adjunct therapies used for clinical management of peri-implant disease?” This was further elaborated using the search terms, “IMPLANT”; “PERI-IMPLANT DISEASE”; “PERI-IMPLANT MUCOSITIS”; “PERI-IMPLANTITIS”; “NON-SURGICAL THERAPY”; “LOCAL THERAPY”; “TOPICAL THERAPY”; “ADJUNCT”. Within a time period ranging from January 2000 to December 2023, clinical studies reporting on the use of local and/or topical adjuncts along with non-surgical therapy for the management of either PIM or PI were included in the search. Studies with sample size less than 10 implants or patients; follow-up period less than 3 months; individual case reports; editorial communications; technical notes and reviews were excluded. The flowchart explaining the literature review process is shown in Figure 11.

Nineteen studies fulfilling the aforementioned criteria were identified and their outcome data were tabulated (Table 1 and Table 2). Out of these, eight studies reported on the management of PIM [8,12,17,18,20,27,28,29], and the remaining 11 studies evaluated local adjuncts used with non-surgical therapy for treatment of PI [6,7,9,10,11,15,16,30,31,32,33]. All studies reported administration of non-surgical therapy along with local adjuncts in the test group that was compared to a suitable control group, except for two studies [11,30], which had no group for comparison. Detailed information pertaining to the specific nature of non-surgical therapy, local adjunct administered in the test and control groups, and outcomes reported are elucidated in Table 1, for peri-implant mucositis, and in Table 2, for peri-implantitis.

## 5. Discussion

Clinical treatment of peri-implant diseases usually involves the sequence of mechanical modalities for the removal of plaque biofilm, followed by sub-mucosal or sub-gingival curettage with specialized tips, which avoid damage to the implant surface [4]. While this comprises non-surgical therapy, additional surgical procedures such as flap surgeries and mechanized implant surface decontamination and polishing (implantoplasty) may be carried out, especially in cases of chronic PI [26]. Irrespective of the nature of professionally delivered therapies for peri-implant hygiene and debridement, patient-centric oral hygiene practices are equally important in controlling infection and inflammation [5,20]. Local application of adjunctive agents has become a routine procedure during peri-implant disease management, because of their ability to provide favorable clinical outcomes, even with simple scaling and minimal non-surgical debridement [1]. Based on the above premise, the present report describes clinical cases with PIM and PI, which were managed with only non-surgical therapy and adjunctive local application of oxygen-based therapy (blue^®^m) gel [23]. This was further reinforced during the treatment phase by advising the patients to follow meticulous oral hygiene with toothpaste and mouthwash of similar oxygen-release formulation (blue^®^m) [24,25]. In order to compare the outcomes and to understand the role of local adjunct therapies in peri-implant disease, a review of the literature was carried out.

The predominant modality of non-surgical therapy in the included studies was ultrasonic scaling, mechanical cleansing with rubber cup and polishing paste, followed by sub-mucosal curettage with specialized instruments, having either a plastic or a Teflon-coated tip, to avoid implant surface damage. In addition, a few studies also reported using either erythritol or glycine in powder form, for sub-gingival air polishing of the implant surface [9,10,11,12]. The procedure of air powder polishing involves spraying a mixture of water and biocompatible abrasive powder to clean and decontaminate the implant surface. This process facilitates the resolution of inflammation and bone gain, around the implant [5]. However, among the studies reporting the use of air abrasion in the present review [9,10,11,12], none of them compared this procedure with other local adjunct therapies. Nevertheless, based on a meta-analysis, Schwarz et al. reported up to a 29.3% reduction in BOP with peri-implant air powder polishing [5]. They further claimed that the use of air abrasion does not significantly add up to the enhancement of clinical outcomes in patients with peri-implant disease, who undergo conventional non-surgical therapy [5]. Thereby implying a definitive role for local adjunct therapies using either antibiotics, antibacterial agents, probiotics, or other biomaterials, in the management of PIM and PI [14,19,34,35].

According to our review, among the different local adjuncts used to treat PIM, chlorhexidine was the most commonly reported agent both in test groups and as a control for comparison. Chlorhexidine formulations such as gels or mouthwashes, and in varying concentrations, were reportedly used as a local adjunct in cases of both PIM and PI. Comparing chlorhexidine gel against a placebo for local administration in PIM, Porras et al. and Heitz-Mayfield et al. reported a significantly improved reduction in inflammation, despite the differing gel concentrations used in the two studies (0.12% and 0.5%, respectively) [27,28]. On the other hand, De Siena et al. compared the local adjunctive effect of chlorhexidine gel (0.2%) against 1% mouthwash in PIM patients and found no difference in outcomes between the two formulations [18]. With respect to adjunctive mouthwashes, a combination of chlorhexidine (0.03%) and cetyl-pyridinium chloride (CPC 0.05%) after non-surgical therapy for PIM, was found to be more effective than using chlorhexidine alone [12]. Similarly, delmopinol hydrochloride (0.2%) mouthwash used as a local adjunct resulted in a greater percentage of patients with disease resolution after PIM, than when chlorhexidine (0.2%) mouthwash was used [20]. In addition to antibacterial agents, amino-acid-buffered sodium hypochlorite gel [29] and probiotics, administered either as lozenges or along with a carrier gel [8,17], were reported based on the review. In both of the above scenarios, there was no complete resolution in inflammatory signs of PIM, although there were significantly improved clinical outcomes [8,17,29].

Among the studies reporting the use of local adjuncts to non-surgical therapy of PI, chlorhexidine was used for comparison in only three studies [16,31,32]. While Machtei et al. reported using sub-gingivally placed chlorhexidine chips as a local adjunct [32], Renvert et al. used chlorhexidine (1%) gel as a control to compare locally applied minocycline microspheres [16]. Chlorhexidine gel administered in the peri-implant tissues using a water-jet irrigation device was tested as an adjunct by Levin et al. [31]. Both local applications of chlorhexidine irrigation and chlorhexidine chips placed sub-gingivally improved PI treatment outcomes such as decreased BOP, reduced PPD, and clinical attachment level (CAL) gain, after three months and six months, respectively [31,32]. Nevertheless, the use of sub-mucosal chlorhexidine gel (1%) did not result in clinical outcomes superior to those achieved with locally administered minocycline microspheres, at 12 months post-treatment [16]. In addition to minocycline, metronidazole, tetracycline, doxycycline, chloramine, and amino-acid-buffered sodium hypochlorite were the other antibiotic or antibacterial agents reported as being used as a local adjunct for PI, among the reviewed studies (Table 2). Invariably, the local use of antibiotics or antibacterials as an adjunct did significantly enhance clinical outcomes, between 3 to 12 months after treatment, compared to when non-surgical therapy was administered alone. Furthermore, sub-gingival application of enamel matrix derivative (EMD) and probiotics administered through oral lozenges were reported as local adjuncts by Kashefimehr et al. [9] and Laleman et al. [10], respectively. Even though EMD application resulted in clinical benefits and the sub-total resolution of inflammation around the implant, probiotics were not clinically effective in enhancing the outcomes of non-surgical therapy for PI [9,10]. Interestingly, none of these studies reported complete clinical resolution.

All three cases being reported herein presented with clinical signs and symptoms of PIM (Case 1) and PI (Cases 2 and 3), which fit with the diagnostic criteria reported in the literature, for the respective peri-implant disease conditions [4]. Similarly, with respect to the non-surgical therapy administered to the patients, they were all in line with what was reported in the included studies for review. Therefore, the nature of local adjunct therapy, based on the TOOTh protocol using blue^®^m formulations [24,25], was the only difference between the cases reported and what was documented in the reviewed literature. The final recall visit was made three months after the last non-surgical therapy procedure (fourth week after the start of treatment), during which time, clinical and radiographic assessments were recorded, to compare with the pre-operative findings.

The clinical and radiographic findings recorded in the presented cases are similar to those that were reported in the literature (Table 1 and Table 2). In addition to significantly enhancing post-operative clinical outcomes such as decreased BOP, no suppuration, reduced PPD, and increased RBL, the use of local oxygen-based therapy (TOOTh protocol) resulted in the near total resolution of PIM and PI, as early as three months after the initiation of therapy (Figure 1, Figure 2, Figure 3, Figure 4, Figure 5, Figure 6, Figure 7, Figure 8, Figure 9 and Figure 10). This may be attributed to the bio-modulatory, anti-inflammatory, angiogenic, and bactericidal effects of local oxygen therapy in wound healing sites [23,24,25,36]. The only other bio-modulatory local adjunct identified through the literature review was the use of probiotics along with non-surgical therapy [8,10,17]. While probiotic administration was shown to improve clinical and immunological benefits of non-surgical therapy for PIM [8,17], the same was not similarly effective for PI [10]. In contrast, local oxygen-based therapy with the blue^®^m formulation resulted in similar favorable clinical and radiographic outcomes for both spectra of peri-implant diseases (PIM and PI).

One of the major limitations of the present report is the small number of cases being reported and the short follow-up times recorded. Nevertheless, these findings shall form the basis for further long-term, multicentric studies. Moreover, the present report demonstrates the ability of clinicians to offer local oxygen-based adjunct therapy along with non-surgical scaling, cleaning, and debridement as a minimally invasive alternative for patients with peri-implant disease, thereby enhancing patient acceptance and compliance. With respect to the review findings reported, there were limitations of heterogeneity in the included study data, mainly about the outcomes assessed and reported, and varying the follow-up periods. Similarly, neither the cases reported, nor the reviewed studies, took into consideration auxiliary modalities such as laser and photodynamic therapy [35], the effect of systemic illnesses on implant osseointegration and bone healing [37,38], and special scenarios such as immediate implant placement and bone graft sites [39].

## 6. Conclusions

Based on the reviewed data and outcomes presented through the clinical cases, it may be concluded that local adjuncts administered along with non-surgical therapy for peri-implant diseases like PIM and PI enhance the clinical outcomes and help in disease resolution. The use of local oxygen-based adjunct therapy (blue^®^m formulations) was equally effective as conventionally reported local adjuncts, such as antibiotics, antibacterials, and probiotics, in decreasing BOP, eliminating suppuration, reducing PPD, and increasing RBL, after non-surgical therapy for PIM and PI. However, the use of local oxygen-based therapy may be considered beneficial in terms of the non-use of topical antibacterials and antibiotics, which are capable of causing hypersensitivity and adverse effects. Nevertheless, future studies comparing local oxygen-based therapy and other local or topical adjuncts, in a long-term clinical setting, are required to design established treatment protocols and prove any plausible efficacy of oxygen-based therapy over conventionally used adjuncts.

## Figures and Tables

**Table 1:** Studies from literature reporting on the use of local adjuncts along with non-surgical therapy for peri-implant mucositis.

Author	Nature of Non-Surgical Therapy	Local Adjunct Therapy	Compared with	Conclusions
Porras et al. [27]	Scaling with plastic scaler + mechanical cleansing with rubber cup and polishing paste	Chlorhexidine (0.12%) gel + rinse (Test)	Placebo (Control)	After 3 months follow-up; use of chlorhexidine (0.12%) gel + rinse as a local adjunct to mechanical therapy for peri-implant mucositis resulted in resolution of inflammation and a significant reduction in PPD.
Heitz-Mayfield et al. [28]	Scaling with plastic scaler + mechanical cleansing with rubber cup and polishing paste	Chlorhexidine (0.5%) gel to be brushed around the implant; twice a day for 4 weeks (Test)	Placebo gel (Control)	After 3 months follow-up; use of chlorhexidine (0.5%) gel as a local adjunct to mechanical therapy did not significantly enhance clinical outcomes in peri-implant mucositis. Implants with restoration margins placed supra-gingivally showed better treatment response than implants with sub-mucosal restoration margins.
De Siena et al. [18]	Professional oral prophylaxis administered by dental hygienist	Chlorhexidine (0.2%) mouthwash 10 mL—rinsed twice a day for 10 days	Chlorhexidine (1%) gel 1 mL placed sub-mucosally twice a day for 10 days	After 3 months follow-up; use of chlorhexidine rinse (0.2%) or gel (1%) as a local adjunct to treat peri-implant mucositis gave better clinical outcomes. Nevertheless; there was no difference in outcomes between the two formulations.
Pulcini et al. [12]	Ultrasonic scaling with plastic tip + erythritol-based air powder polishing	Chlorhexidine (0.03%) + CPC (0.05%) mouthwash (Test)	Mouthwash without chlorhexidine or CPC (Control)	After 12 months follow-up; use of chlorhexidine (0.03%) + CPC (0.05%) mouthwash as a local adjunct in peri-implant mucositis resulted in better clinical outcomes than with mouthwash without the above active ingredients. However, the formulation did not result in complete resolution of peri-implant disease.
Iorio-Siciliano et al. [29]	Ultrasonic scaling with plastic tips + mechanical cleansing with rubber cup and polishing paste	Amino acid buffered sodium hypochlorite gel—applied 5 times in the peri-implant tissues immediately after non-surgical therapy (Test)	Placebo gel—applied in the same way as test group (Control)	After 6 months follow-up; use of sodium hypochlorite gel as a local adjunct to non-surgical therapy of peri-implant mucositis resulted in a significant reduction in PPD and number of implants with BOP, which was better than that with placebo gel, but not significantly. Neither modality resulted in complete peri-implant disease resolution.
Philip et al. [20]	Ultrasonic scaling with plastic tips + mechanical cleansing with rubber cup and polishing paste	Delmopinol hydrochloride (0.2%) mouthwash twice daily until follow-up (Test)	Chlorhexidine (0.2%) mouthwash twice daily until follow-up (Positive Control)/Placebo mouthwash twice daily until follow-up (Negative Control)	After 3 months follow-up; use of delmopinol hydrochloride mouthwash as an adjunct to non-surgical therapy of peri-implant mucositis resulted in a significant improvement in clinical parameters, than with the use of chlorhexidine mouthwash. There was 87% disease resolution among patients who used delmopinol mouthwash; in comparison to 60% and 71% in those who used chlorhexidine and placebo mouthwashes, respectively.
Alqahtani et al. [17]	Ultrasonic scaling with plastic tips + mechanical cleansing with rubber cup and polishing paste	Probiotic lozenge containing *Lactobacillus reuteri*; chewed orally twice a day after brushing; for 21 days (Test)	Amoxycillin 500 mg administered orally; three times a day for 7 days (Positive control)/Non-surgical therapy only (Negative control)	After 3 months follow-up; use of probiotic therapy as a topical adjunct to non-surgical therapy of peri-implant mucositis was more effective than adjunct antibiotic therapy in terms of significantly improved clinical outcomes.
Santana et al. [8]	Ultrasonic scaling with Teflon-coated tips + mechanical cleansing with rubber cup and polishing paste	Topically applied carboxymethyl cellulose gel containing a probiotic formulation of *Bifidobacterium lactis*, *Lactobacillus rhamnosus*, and *Lactobacillus paracasei* (Test)	Non-surgical therapy only (Control)	After 6 months follow-up; use of probiotic therapy as a topical adjunct to non-surgical therapy of peri-implant mucositis in edentulous patients resulted in significantly improved clinical outcomes and immunological benefits.

PPD—Probing pocket depth; CPC—Cetylpyridinium chloride BOP—Bleeding on probing.

**Table 2:** Studies from literature reporting on the use of local adjuncts along with non-surgical therapy for peri-implantitis.

Author	Nature of Non-Surgical Therapy	Local Adjunct Therapy	Compared with	Conclusions
Mombelli et al. [30]	Scaling with plastic scaler + mechanical cleansing with rubber cup and polishing paste	Tetracycline fibers were placed in pocket for 10 days	-	After 6 months follow-up; use of tetracycline as a local adjunct to non-surgical therapy of peri-implantitis resulted in a significant improvement in clinical parameters and reduction in microbial colonies.
Renvert et al. [16]	Scaling with plastic scaler + mechanical cleansing with rubber cup and polishing paste	Minocycline microspheres (1 mg) placed sub-mucosally (Test)	Chlorhexidine (1%) gel 1 mL placed sub-mucosally (Control)	After 12 months follow-up; use of minocycline as a local adjunct to mechanical therapy for peri-implantitis resulted in a greater sustained reduction in PPD over 12 months, than with the use of chlorhexidine.
Levin et al. [31]	Ultrasonic scaling and surface debridement with specialized instruments	Water jet irrigation with chlorhexidine gel 5 mL (Test)	Only water jet irrigation (Control)	After 3 months follow-up; use of local chlorhexidine gel delivered through water jet irrigation as an adjunct to mechanical therapy for peri-implantitis significantly decreased BOP and PPD, than when using water jet alone. There was no significant improvement in RBL in both groups.
Roos-Jansåker et al. [6]	Ultrasonic scaling with sub-mucosal debridement using piezo-ceramic scaler tips	Sub-mucosally administered chloramine to cover all implant surfaces (Test)	Only scaling and debridement (Control)	After 3 months follow-up; use of local chloramine as an adjunct to non-surgical therapy of peri-implantitis was only as effective as conventional treatment. Irrespective of the use of chloramine or not, there was a significant improvement in clinical outcomes.
Kashefimehr et al. [9]	Sub-gingival scaling with plastic tips + air polishing with glycine-based powder	EMD administered sub-mucosally; 2 weeks after non-surgical therapy (Test)	Non-surgical therapy only (Control)	After 3 months follow-up; use of EMD as a local adjunct to non-surgical mechanical therapy for peri-implantitis resulted in a significant improvement in clinical outcomes, in comparison to mechanical debridement alone. There was no complete disease resolution either with or without EMD.
Mensi et al. [11]	Ultrasonic scaling + supra- and sub-gingival erythritol-based air powder polishing	Doxycycline administered supra- and sub-gingivally (one week after non-surgical therapy + additional peri-implant doxycycline application one week later)	-	After 12 months follow-up; use of multiple anti-infective adjunct therapy with doxycycline and eythritol air polishing along with mechanical therapy for peri-implantitis resulted in a significant improvement in clinical parameters.
Laleman et al. [10]	Ultrasonic scaling with specialized tips + sub-gingival debridement with titanium curettes + Air polishing	Dual strain probiotic *Lactobacillus reuteri* drops in peri-implant area after non-surgical therapy + lozenges (1–2 per day) containing the above probiotic strains for 12 weeks (Test)	Placebo drops and lozenges without probiotic bacteria (Control)	After 6 months follow-up; use of dual strain probiotic *Lactobacillus reuteri* as an adjunct for non-surgical therapy of peri-implantitis showed no clinically discernible benefits.
Mayer et al. [7]	Ultrasonic scaling with specialized tips + sub-gingival debridement with Teflon-coated curettes	Amino acid buffered sodium hypochlorite gel—applied 3 times in the peri-implant tissues immediately after non-surgical therapy + 1 mg minocycline (Test)	Non-surgical therapy only (Control)	After 12 months follow-up; use of sodium hypochlorite gel with minocycline as a local adjunct to non-surgical therapy of peri-implantitis resulted in a significant reduction in inflammation and better connective tissue reattachment. This formulation provided a local antiseptic and anti-inflammatory effect.
Machtei et al. [32]	Supra-gingival ultrasonic scaling + sub-gingival implant surface debridement with specialized tips	Sub-gingival chlorhexidine chips applied bi-weekly for 12 weeks (Test)	Non-surgical therapy only (Control)	After 6 months follow-up; use of chlorhexidine chips as a local adjunct to non-surgical therapy of peri-implantitis resulted in a significant improvement in clinical parameters (PPD and CAL).
Park et al. [33]	Ultrasonic scaling + sub-gingival mechanical debridement with specialized tips	Metronidazole + Minocycline ointment administered locally (Test 1)/Minocycline ointment administered locally (Test 2)	Non-surgical therapy only (Control)	After 3 months follow-up; use of either a combination of metronidazole and minocycline or minocycline alone as a local adjunct to non-surgical therapy of peri-implantitis resulted in significantly improved clinical treatment outcomes. However, in deep pockets (≥8 mm), the use of metronidazole and minocycline resulted in greater PPD reduction.
Alhumaidan et al. [15]	Ultrasonic scaling + sub-gingival mechanical debridement with specialized tips	Minocycline microspheres (1 mg) placed sub-gingivally (Test)	Non-surgical therapy only (Control)	After 6 months follow-up; use of minocycline administered sub-gingivally as a single-use adjunct to non-surgical therapy of peri-implantitis resulted in significantly improved clinical outcomes than with the use of non-surgical therapy alone. It may be assumed that only topical application of minocycline in peri-implantitis might be as effective as non-surgical therapy alone.

PPD—Probing pocket depth; BOP—Bleeding on probing; EMD—Enamel matrix derivative; CAL—Clinical attachment level; RBL—Radiographic bone level.

## Data Availability

All data are contained within the article.
